# The F1Fo-ATPase inhibitor protein IF1 in pathophysiology

**DOI:** 10.3389/fphys.2022.917203

**Published:** 2022-08-04

**Authors:** Cristina Gatto, Martina Grandi, Giancarlo Solaini, Alessandra Baracca, Valentina Giorgio

**Affiliations:** Department of Biomedical and Neuromotor Sciences, University of Bologna, Bologna, Italy

**Keywords:** inhibitor protein IF1, mitochondria, cancer, neurodegeneration, ATP synthase

## Abstract

The endogenous inhibitor of ATP synthase is a protein of about 10 kDa, known as IF1 which binds to the catalytic domain of the enzyme during ATP hydrolysis. The main role of IF1 consists of limiting ATP dissipation under condition of severe oxygen deprivation or in the presence of dysfunctions of mitochondrial respiratory complexes, causing a collapse in mitochondrial membrane potential and therefore ATP hydrolysis. New roles of IF1 are emerging in the fields of cancer and neurodegeneration. Its high expression levels in tumor tissues have been associated with different roles favouring tumor formation, progression and evasion. Since discordant mechanisms of action have been proposed for IF1 in tumors, it is of the utmost importance to clarify them in the prospective of defining novel approaches for cancer therapy. Other IF1 functions, including its involvement in mitophagy, may be protective for neurodegenerative and aging-related diseases. In the present review we aim to clarify and discuss the emerging mechanisms in which IF1 is involved, providing a critical view of the discordant findings in the literature.

## Introduction

The natural inhibitor of the mitochondrial F1Fo ATPase is known as IF1, a protein of about 10 kDa identified in 1963 (Pullman and Monroy, 1963), which has been characterized in detail for its molecular structure and activity (Cabezon et al., 2000; Cabezón et al., 2001; Jonathan R, 2003; Gledhill et al., 2007). The inhibitory protein has been described in mammals, flies, plants, and yeast, although size and sequence of the IF1 homologous show variations (Cintrón and Pedersen, 1979; Norling et al., 1990; Harris and Das, 1991; Cabezón et al., 2002; Ichikawa et al., 2006). IF1 binds to the F1 catalytic domain of the ATP synthase complex, when the enzyme works in reverse. It is known to be a reversible non-competitive inhibitor of ATP hydrolysis (Green and Grover, 2000). The role of the inhibitor protein IF1 consists of limiting ATP dissipation, under conditions of severe or complete oxygen deprivation, as seen in heart ischemia (Rouslin and Pullman, 1987; Solaini and Harris, 2005; Campanella et al., 2009). When the mitochondrial membrane potential (ΔΨ) is compromised, during oxygen deprivation or damage of mitochondrial respiratory complexes (associated or not with mutations of specific genes), the thermodynamic equilibrium favours the reversal activity of the ATP synthase which works hydrolysing ATP. The enzyme may then act as a proton motive ATPase (Zanotti et al., 1987; Jennings et al., 1991) consuming ATP and translocating protons from the mitochondrial matrix to the intermembrane space (Solaini and Harris, 2005). IF1 binding to the isolated F1Fo complex and inhibition of ATP hydrolysis are favoured by an acidic pH, with optimal inhibition occurring between pH 6.5–6.7 (Cabezon et al., 2000), Figure 1. On the other hand, the release of the inhibitor protein occurs at higher pH and in the presence of mitochondrial membrane potential (Power et al., 1983; Panchenko and Vinogradov, 1985). The N-terminal region of the bovine inhibitor IF1, consisting of residues 1–46, was shown to be responsible of ATPase activity inhibition through the binding to one of the three catalytic interfaces of bovine F1-ATPase structure (Jonathan R Gledhill et al., 2007). The critical N-terminal residues of IF1 (E30, Y33, F34, Q41, L42) that are involved in the initial interaction with the F1 sector, were identified by point mutation studies (Bason et al., 2011). Recently, kinetic analysis of the IF1 binding confirmed the role of Y33 in the initial association and showed that the E30A mutation destabilizes the locked state without affecting formation of the initial IF1-F1 complex (Kobayashi et al., 2021).

**FIGURE 1:**
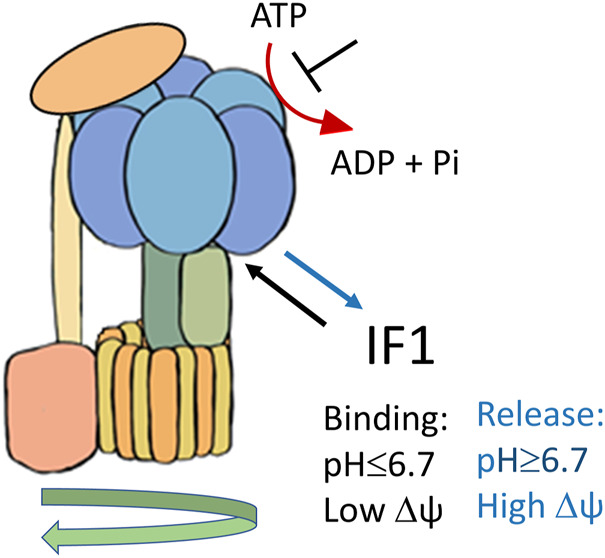
Schematic representation of IF1 binding to F1Fo-ATPase, favoured by acidic pH and membrane potential (Δψ) collapse.

In heart ischemia, IF1 binding to the F1 complex of the enzyme may inhibit the ATPase activity limiting the ATP decrease only to a 30–50% (Jennings et al., 1991; Solaini and Harris, 2005). This allows cellular ATP preservation (Atwal et al., 2004; Di Pancrazio et al., 2004), thus avoiding necrotic damage of anoxic tissues. In addition, IF1 plays a role in heart preconditioning since it limits the damage caused by prolonged ischemia (Bosetti et al., 2000). This occurs because it has been shown that repeated short ischemic insults modulate the binding between F1 and the inhibitor IF1 during the early phase of reperfusion (Bosetti et al., 2000), thus pre-adapting the heart to a long anoxia experience, and limiting necrosis.

Substantial differences in the intracellular levels of IF1 are reported between different tissues and cell types within tissues. Therefore, it turns out that the IF1 levels might be important in the mechanisms underlying tissue-specific vulnerability to hypoxic injury. IF1 expression analysis in human heart, kidney and liver showed that their IF1 mRNA levels are similar, but essentially doubled those of the brain tissues. The IF1 content in heart and brain was four times that of the ATP synthase, whereas in liver and kidney, the ATP synthase content exceeded that of IF1. On the contrary, very low IF1 protein levels were detected in mouse heart and liver, underlining differences in IF1 expression between species (Rouslin et al., 1995; Esparza-Molto et al., 2019).

The interaction of IF1 dimers with dimeric ATP synthase complexes have been resolved in a recent cryo-EM structure (Gu et al., 2019). Self-association of IF1 dimers in tetramers involves the N-terminus of the protein hence masking its inhibitory region. Protonation/deprotonation of the key residue H49 modulates tetramer formation (Cabezon et al., 2000). The equilibrium between dimers and inactive tetramers of IF1 is crucial in the *in vivo* modulation of the inhibitory activity and is sensitive to IF1 concentration, pH and ions fluctuations (Boreikaite et al., 2019).

In addition, it has been proposed that IF1 levels circulating in the extracellular space modulate important signalling pathways, that involve the ectopic form of ATP synthase (Gore et al., 2022). Ecto-ATP synthase was defined when the entire monomeric F1Fo ATP synthase complex, containing both the nuclear and the mitochondrial DNA-encoded subunits, was found on the plasma membrane (Bae et al., 2004; Mangiullo et al., 2008; Giorgio et al., 2010; Rai et al., 2013). Under physiological conditions ecto-ATP synthase was shown to hydrolyse ATP in the extracellular space, thus affecting the external ATP/ADP levels (Martinez et al., 2003; Mangiullo et al., 2008). Endogenous IF1 has been revealed on the plasma membrane of hepatocytes (Contessi et al., 2007) and endothelial cells (Cortés-Hernández et al., 2005) and it was shown to bind to, and modulate, the ecto-ATP synthase by inhibiting its hydrolase activity (Giorgio et al., 2010). The latter findings suggest the involvement of IF1 in the signalling pathways mediated by ecto-ATP synthase, taking place in hepatocytes, endothelial and cancer cells (Radojkovic et al., 2009; Lichtenstein et al., 2015; Gore et al., 2022). In hepatocytes IF1 regulates the HDL endocytosis by modulating, through the inhibition of extracellular ATP hydrolysis, the ADP-responsive P2Y receptor pathway (Cardouat et al., 2017). In endothelial cells the P2Y receptor activation by ATP hydrolysis was shown to promote cell proliferation through a PI3K/β-Akt signalling pathway (Castaing-Berthou et al., 2017). On the same line, ectopic IF1 was suggested to limit ATP hydrolysis and therefore proliferation in lung and breast cancers and myeloid leukaemia cells (Pan et al., 2011; Chang et al., 2012; Zhao et al., 2012). However, given the focus of this review on mitochondria, details on these aspects can be found in another recent review by Emilia Gore and coworkers (Gore et al., 2022).

### The mitochondrial protein IF1 in cancer

**FIGURE 2:**
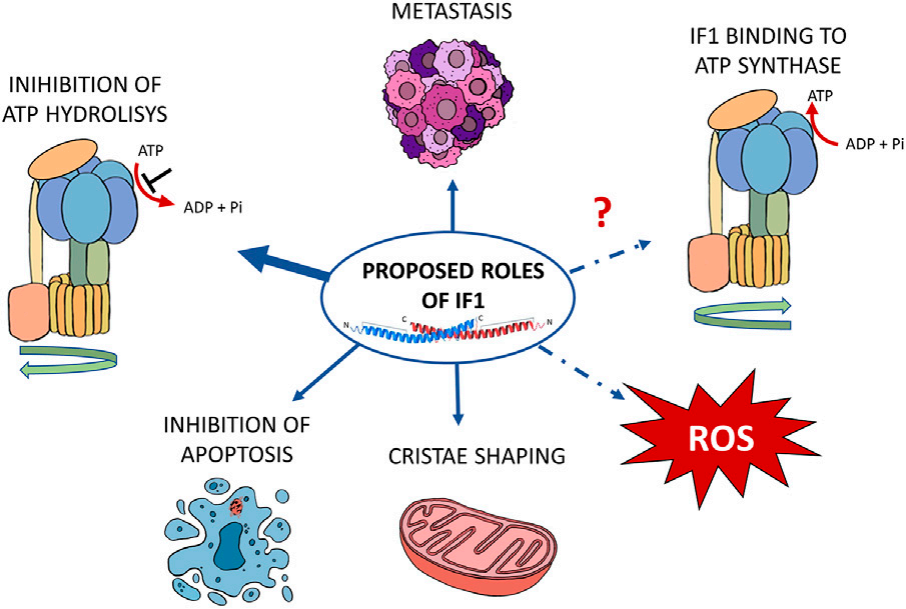
Schematic representation of the proposed functions of IF1 in cancer. According to the literature, IF1 affects cancer development through its effects on metastasis and cristae shaping, inhibition of ATP hydrolysis and of apoptosis. A possible role has also been proposed in the modulation of both ROS level and ATP synthase during oxidative phosphorylation. The proposed mechanisms are indicated by blue arrows. The IF1 roles that are still under investigation are indicated by the dashed lines.

IF1 was shown to play a role in promoting cancer development and growth (Torresano et al., 2020; Solaini et al., 2021). Its high expression was described in human tissues from ovarian, colon, lung and breast tumors (Sánchez-Aragó et al., 2013). Different mechanisms favouring tumor formation have been proposed for this small mitochondrial protein, depending on the metabolic conditions of the different tumors (Figure 2).

### IF1 in hypoxic tumors and ATP hydrolysis

It is well known that many cancers adopt the so-called aerobic glycolysis, first described by Otto Warburg (Warburg, 1956), in which glycolysis and mitochondrial oxidative phosphorylation co-exist, supporting the energy requirement for cell proliferation. Transient oxygen fluctuations in solid tumors can cause hypoxia/anoxia and therefore ATP hydrolysis, which occurs in mitochondria. Although demonstrations *in vivo* are not available on the role of IF1 in the survival and development of hypoxic/anoxic tumors, clear-cut results have been obtained *in vitro* by studies carried out in IF1 silenced osteosarcoma cells under anoxia-mimicking conditions (Sgarbi et al., 2018a). The ATP hydrolysis was completely inhibited by IF1 in the osteosarcoma control cells, and IF1 favoured survival and proliferation of near-anoxic/anoxic cells, preventing ATP dissipation. This result matches previous observations in isolated mitochondria from controls and IF1-silenced cells (Barbato et al., 2015). In addition, although ROS levels decrease in non-transformed cells under hypoxia (Sgarbi et al., 2017), a further decrease was observed in IF1 expressing tumor cells (Sgarbi et al., 2018a). Studies on osteosarcoma 143B and liver carcinoma HepG2 cell models showed that both cell lines exhibited increased glycolysis when exposed to hypoxia (Chevrollier et al., 2005; Sgarbi et al., 2018a). Chevrollier and coworkers suggested that ATP hydrolysis by the F1 sector of ATP synthase is allowed by glycolytic ATP uptake into mitochondria of the aforementioned cellular models under oxygen deprivation, through the adenine nucleotide transporter (ANT). In fact, HepG2 cells expressing lower ANT levels of those in 143B cells were subjected to a cell cycle arrest in G1 phase, showing higher sensitivity to hypoxia compared to the osteosarcoma model (Chevrollier et al., 2005). This highlights the importance of ATP hydrolysis in cell proliferation, and thus the role of IF1 in the inhibition of this process. Similarly, the pancreatic ductal adenocarcinoma PDAC cell lines, showing a higher IF1 content and IF1/ATP synthase ratio than pancreatic acinar cells PACs, exhibited the ability of maintaining their ATP levels under conditions of chemical hypoxia (Tanton et al., 2018). The rate of ATP hydrolysis was also inhibited by IF1 in intact mitochondria from hepatoma after preincubation with an uncoupler (Chernyak et al., 1991). Interestingly, these authors suggested that in energized hepatoma mitochondria IF1 was bound to ATP synthase in a non-inhibitory site, an important indication which requires further investigation.

### IF1 in normoxic tumors and ATP synthesis

Recent findings suggest that IF1 might also bind to ATP synthase when the enzyme works physiologically and synthesizes ATP (Barbato et al., 2015; García-Bermúdez et al., 2015; Kahancová et al., 2018). However, a mechanism (or site) promoting the IF1 interaction with the enzyme during ATP synthesis remains to be investigated, since the binding of the IF1 N-terminal domain to the ATP synthase catalytic subunits (α/β) require sequential hydrolysis of two ATP molecules (Bason et al., 2014; Kobayashi et al., 2021).

Discordant results have been published concerning the possible regulation of the oxidative phosphorylation by IF1. Indeed, in osteosarcoma model, IF1 expression slightly increased the oxidative phosphorylation rate compared to IF1-silenced cells (Barbato et al., 2015). On the contrary, it was proposed that IF1 binds to the ATP synthase and inhibits ATP synthesis (García-Bermúdez et al., 2015). The latter authors proposed that a post-translational regulation of IF1, which is affected by the metabolic state of cells, mediates the inhibitor protein binding to the enzyme. They reported a S39 phosphorylation of the human inhibitor protein, by a mitochondrial cAMP-dependent protein kinase, which disfavours the IF1 interaction with ATP synthase (García-Bermúdez et al., 2015).

**FIGURE 3:**
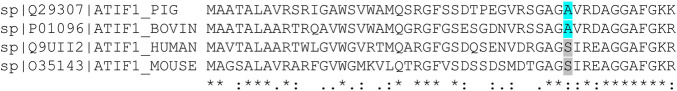
Multiple alignment of the amino acid sequence (residues 1–50) of human IF1 with equivalent portions of F1-ATPase inhibitor from pig, bovine, mouse proteins. The A39 residues are in light-blue in porcine and bovine, while S39 residues are grey in human and mouse sequences. The UniProt accession numbers for inhibitor proteins from *Sus Scrofa*, *Bos taurus*, *Homo sapiens*, *Mus musculus*, are shown on the left of each sequence. Asterisk (*) indicates positions which have a single, fully conserved residue; colon (:) indicates conservation between amino acid groups or similar properties.

However, human S39 is replaced by alanine in porcine and bovine IF1 sequences (Figure 3), (Bason et al., 2011), while it is conserved in rodents (Bason et al., 2011; Esparza-Molto et al., 2019). Although the literature offers many examples that only partly conserved AA residue can still work as a structural/functional residue, and the S39 phosphorylation has been previously reported at a proteomic level (Zhao et al., 2011; Sharma et al., 2014), its physiological role has only been proposed by (García-Bermúdez et al., 2015). Moreover, it was proposed that NFκB pathway and cell proliferation are promoted in cancer by increased ROS levels, that are produced through an electron backflow at the level of the mitochondrial respiratory chain in a condition in which the ATP synthase is inhibited by the inhibitor protein IF1 (Sánchez-Aragó et al., 2013). Although these findings found support by experiments in cultured cells overexpressing or downregulating IF1 (Sánchez-Cenizo et al., 2010), there is intense debate on the latter specific functions of IF1 in the literature (see the recent review by Solaini et al., 2021). The inhibition of ATP synthesis by the inhibitor protein was not matched by the majority of studies reporting measurements of ATP synthesis rate and ΔΨ both in IF1-silenced and control cancer cells (Fujikawa et al., 2012; Runswick et al., 2013; Barbato et al., 2015; Sgarbi et al., 2018a; Tanton et al., 2018). Furthermore, a decrease in ROS formation was described in osteosarcoma cells in which IF1 is stably silenced (Sgarbi et al., 2018b), in line with a slightly decreased state 3 respiration upon IF1 downregulation (Barbato et al., 2015). These findings are not due to a different ATP synthase oligomer stability, nor to modified levels of the respiratory chain complexes, as revealed by electrophoretic analysis followed by western blotting and protein immunodetection (Barbato et al., 2015). Nevertheless, they might be explained by the role of IF1 in keeping mitochondrial cristae morphology (Campanella et al., 2008), as detailed in the following paragraph. Notably, the disclosure of a different binding site/mechanism of IF1 under oxidative phosphorylation conditions (i.e. ATP synthesis) might be of the utmost importance to clarify the aforementioned discussion on the inhibitor protein mechanisms of action in normoxic tumors.

### The role of IF1 in mitochondrial morphology and apoptosis

Mitochondrial morphology and structure prevent the activation of the apoptotic pathways by sequestering cytochrome c (Cogliati et al., 2016). A rearrangement of cristae organization is required during apoptosis to allow the cytochrome c release (Cogliati et al., 2016). ATP synthase dimer stability is one of the essential factors affecting cristae ultrastructure (Strauss et al., 2008; Cogliati et al., 2016). In support of this hypothesis, it has recently been observed that dimer dismantling upon the downregulation of the ATP synthase f subunit causes changes in mitochondrial cristae morphology of human cells (Galber et al., 2021). Moreover, IF1 silencing causes a decrease in cristae density, while its upregulation has the opposite effect, highlighting a role of IF1 in mitochondrial cristae organization (Campanella et al., 2008; Faccenda et al., 2013). Consistent with the role of the inhibitor protein in stabilizing cristae morphology, cytochrome c release was delayed in IF1 overexpressing cells, thus limiting cellular apoptosis (Faccenda et al., 2013). As proposed, IF1 can contribute to the maintenance of mitochondrial cristae ultrastructure by two mechanisms: 1) stabilization of ATP synthase oligomers (García et al., 2006; Campanella et al., 2008; Spikes et al., 2021) and 2) inhibition of OMA1-dependent proteolytic cleavage of the dynamin GTPase optic atrophy 1 (OPA1), thus preventing cristae remodeling that occurs during apoptosis (Faccenda et al., 2017). Furthermore, IF1 silencing promotes mitochondrial Ca^2+^ overload through alteration of cristae morphology and increased activity of the mitochondrial Ca^2+^ uniporter (Faccenda et al., 2021). Recent findings showed that the expression of the inhibitor protein carrying the H49K mutation, which impedes IF1 dimers self-association into tetramers, led to alterations of the organization of ATP synthase slightly increasing the enzyme oligomeric states, in association with changes in mitochondrial cristae ultrastructure (Weissert et al., 2021).

Although some studies have confirmed the role of IF1 in ATP synthase oligomer stability, other fail to agree on the proposed mechanism in mitochondrial cristae remodelling. The stability ATP synthase oligomer/dimer stability (Kahancová et al., 2018) or mitochondrial morphology were unchanged in cells in which the IF1 protein was either knocked down (Fujikawa et al., 2012; Barbato et al., 2015) or overexpressed (Kahancová et al., 2020). Overall, the effect of IF1-mediated ATP synthase oligomers on cristae ultrastructure is controversial.

The protective role of IF1 in promoting resistance to apoptosis in pathological conditions associated with OXPHOS deficiency also remains controversial. The maintenance of Δψ at the expense of ATP in IF1-silenced mouse hepatocytes treated with antimycin A, a complex III inhibitor, protected cells from death (Chen et al., 2014). Moreover, Rho0 cells, lacking mitochondrial DNA and having impaired OXPHOS (Chandel and Schumacker, 1999), were found to be resistant to apoptosis (Higuchi et al., 1997). The resistance to apoptotic stimuli of Rho0 cells cannot be attributed to ATP synthase dimer stability (Wittig et al., 2010), creating the potential for investigating other molecular mechanisms at the basis of IF1 involvement in protection from death. This seems particularly interesting for those cancer models in which a high level of IF1 might control apoptosis. Although the role of IF1 in the inhibition of apoptosis has been proposed in cancer, its effect needs to be clarified on the modulation of the mitochondrial permeability transition pore, the mitochondrial mega-channel which is desensitized in tumor models and leads to cell death (Galber et al., 2020).

### IF1 and the metastatic behavior of tumors

The inhibitor protein is largely upregulated in some cancers, as shown in lung, colon, breast and ovarian carcinomas, where it has been identified as a prognostic marker for patients, since its expression level is very low in the corresponding healthy tissues. This IF1 upregulation in many cancers has been proposed to be important for cell adaptation to transient metabolic requirements (Formentini et al., 2012; Sánchez-Aragó et al., 2013).

By contrast, human tissues such as liver, stomach, kidney and endometrium that physiologically have high inhibitor protein levels, do not increase the IF1 content during cancer development (Sánchez-Aragó et al., 2013; Esparza-Moltó et al., 2017). Although different functions have been proposed for IF1 in the primary tumor development, its role in metastasis and migration remains unclear (Table 1). High IF1 levels are associated to a worse patient prognosis in bladder carcinomas (Wei et al., 2015), gliomas (Wu et al., 2015) and non-small-cell lung cancer (Gao et al., 2016). A poor prognosis is also accompanied by high IF1 levels in hepatocarcinoma (Song et al., 2014) and stomach cancer (Yin et al., 2015). On the same line, both the downregulation and knockout of IF1 in the PANC-1 pancreatic cancer cells reduced migration, invasion and proliferation, supporting the role of IF1 in growth and metastasis of patients pancreatic ductal adenocarcinoma (Tanton et al., 2018). Importantly, in the latter cellular model it was shown that, in the absence of the inhibitor protein, a drop in mitochondrial membrane potential was observed when cells were subjected to anoxia-mimicking condition (Tanton et al., 2018).

**TABLE 1:** The effects of IF1 expression levels on tumor growth, migration and metastasis in different models.

More aggressive tumors with high IF1	Tumor Tissue/Cells	References
• IF1 expression is low in normal lung tissues, increases in early stages of NSCLC, and reaches the highest levels in advanced tumor progression	• NSCLC biopsies (divided in lung adenocarcinoma, squamous cell carcinoma and large-cell carcinoma)	• Gao et al., 2016
• IF1 is overexpressed in Bladder cancer tissues and cells	• Bladder cancer and normal cell lines	• Wei et al., 2015
IF1 KD induces inhibition of cell growth via cell cycle arrest at the G0/G1 phase and inhibition of migration		
• IF1 expression is significantly increased in glioma tissues compared with the normal tissues	• Glioma brain tissues Glioma cell lines (U251, U87)	• Wu et al., 2015
IF1 KD inhibits glioma cell migration and invasion		
IF1 KD shows E-cadherin upregulation and Snai1 and NF-kB inhibition		
• *In vivo* model: mice injected with SMMC7721-IF1 cell line, expressing high IF1 levels, show more and bigger lung metastases. Mice injected with HCCLM3- shRNA-IF1 cells (IF1 KD) had less and smaller lung metastases	• Hepatocarcinoma biopsies Hepatocarcinoma cell lines mouse xenografts	• Song et al., 2014
Mouse xenografts show that the decreased IF1 expression limits HCC-induced angiogenesis		
Cell models overexpressing IF1 promote Snai1 and VEGF expression by activating NFkB signaling		
IF1 expression increases in advanced-stage liver cancer compared to normal tissue		
Overexpression of IF1 significantly increases the migratory and invasive cells behaviour		
IF1 KD in the highly invasive HCCLM3 and MHCC-97H cells decreases their migratory and invasive behaviors		
IF1 induces EMT in HCC cells by the repression of E-cadherin and β-catenin and increases the expression of vimentin, fibronectin and Snai1		
• IF1 expression in gastric cancer tissues is significantly higher than in normal tissues	• Gastric cancer tissue Gastric cancer cell line	• Yin T et al., 2015
IF1 KD inhibits cell proliferation and induces apoptosis	BALB/c nude mice injected with SGC-7901 cells	
*In vivo* model: IF1 KD slows down tumor growth in tumor bearing mice		
IF1 KD reduces the number of Ki-67 positive tumor cells and increases the number of TUNEL positive tumor cells		
IF1 KD decreases the migratory and invasive cells behaviour		
• High IF1 level in tumor tissues	• Lung cancer Lung cancer cell lines (HLRT104 HOP62, A549)	• Sánchez-Aragó et al., 2013
IF1 overexpression increases ROS production and this protects the cell from STS-induced cell death		
IF1 overexpression increases the activation of NF-kB promoter by a decreasing of ikBα		

Less aggressive tumors with high IF1	Tumor Tissue/Cells	References
• High IF1 level in the tumor tissues	• Breast and colon cancer biopsies Breast and colon cancer cell lines	• Sánchez-Aragó et al., 2013
• Transcriptomic analysis shows that IF1 overexpressing cells have less aggressive phenotype	• Breast cancer biopsies Triple-negative breast cancer cell line (BT549-luc)	• García-Ledo et al., 2017
IF1 overexpression supports the maintenance of ECM and tissue integrity		
IF1 overexpression increase the E-Cadherin levels and decrease the integrin β1 levels		
IF1 overexpression shows less growth capacity and migration potential		
• IF1 decreases from the primary tumor to the lymph node metastases	• Primary breast cancer and synchronous lymph node metastases (LNM) biopsies, primary breast cancer and the distant metastasis (DM) biopsies	• Kurbasic et al., 2015
• Colon cancer cells with low IF1 expression have a more pro-oncogenic phenotype with increased migratory and invasive capabilities and increased capacity to evade death than IF1 overexpressing cells	• Colorectal adenocarcinoma biopsies Colorectal adenocarcinoma cell line (HCT116)Mouse xenograft injected with shIF1 or IF1 HCT116 cells	• Gonzales Llorente et al., 2020
shIF1 cells have a deregulation of cell cycle and an inhibition of proliferation due to the inhibition of the PPAR signaling pathway		
*In vivo* model: low expression of IF1 confers an increased metastatic potential to lung bigger dimension of secondary tumors		
Decreased NK cell infiltration and resulting cell-death in shIF1-colonospheres contribute to the metastatic potential of shIF1 cells	• Gastric cancer cell lines	
• Analysis of gastric cancer dataset shows IF1 high levels and correlates with a better prognosis	• Gastric cancer dataset	• Zhang et al., 2016

In contrast, a better prognosis is predicted in breast and colon cancer patients expressing high IF1 tissue levels (Sánchez-Aragó et al., 2013; Zhang et al., 2016; González-Llorente et al., 2020), especially in the case of triple-negative breast cancer (García-Ledo et al., 2017). Concerning breast cancer, it was shown that lymph node metastases express lower levels of the inhibitor protein compared to primary tumor (Kurbasic et al., 2015). Moreover, a recent study described that low IF1 levels conferred a metastatic phenotype to a triple-negative breast cancer model, in line with the hypothesis that in breast cancer lower IF1 expression levels might determine a highly invasive potential (García-Ledo et al., 2017). Accordingly, oxidative phosphorylation was higher in metastasis derived from 4T1 mouse adenocarcinoma cells implanted *in vivo* if compared to that in the corresponding primary tumors (LeBleu et al., 2014). Although the underlining mechanisms of IF1 overexpression in tumor invasion and metastasis have not yet been elucidated, tissue-specific functions of IF1 might be hypothesized. A plausible IF1 mechanism might be related to the different metabolic requirements and adaptations of the different cancer types. On the one hand, high IF1 levels might guarantee cell survival avoiding ATP dissipation in solid tumors, where cells experience hypoxic conditions and anoxia, as in the aforementioned pancreatic cancer, on the other, high IF1 expression levels might disfavour cancer invasion and progression in those normoxic tumors which require active oxidative phosphorylation for metastasis.

### Does mitochondrial IF1 affects neuronal diseases or neurodegeneration?

The neurodegenerative diseases (Alzheimer’s disease, Parkinson’s disease, Huntington disease and amyotrophic lateral sclerosis) are age-related, and mitochondrial dysfunction is often associated to the progression of these diseases (Bosetti et al., 2002; Squitieri et al., 2006; Celsi et al., 2009). There is little knowledge on the role of the inhibitor protein IF1 in the onset of neurodegenerative diseases. Neurons are highly energy dependent cells, therefore dysfunctions of mitochondrial OXPHOS can result in both reduced ATP and increased ROS production leading to oxidative stress and Ca^2+^ deregulation. Neurons are highly oxidative, while astrocytes are more dependent on glycolysis (Peuchen et al., 1996; Magistretti, 2006). The IF1 levels relative to the ATPase *ß* subunit are significantly higher in neurons than in astrocytes (Campanella et al., 2008). The functional impact due to the difference in IF1 expression was assessed by inhibiting respiration with NaCN in the two cellular types. The NaCN treatment caused the progressive loss of ΔΨ in neurons, but not in astrocytes, indicating active ATP hydrolysis in astrocytes (Campanella et al., 2008). The same authors suggested a protective role of IF1 against cell death induced by oxygen and glucose deprivation, proposing that IF1 expression may have a significant effect on ischemic injury. Upregulation of IF1 levels was shown in neurons under hypoxia/ischemia conditions, thus pointing toward a role of IF1 in preservation and protection of cortical neurons and the neuronal cell line SH-SY5Y (Matic et al., 2016). The neuroprotective effect of IF1 was attributed to its role in mitophagy, through the induction of PINK-1 accumulation and the recruitment of the mitophagic ubiquitin ligase PARK-2, in order to recycle the mitochondrial population (Matic et al., 2016). On the same line, the pharmacological compound BTB06584 was proposed to inhibit ATP hydrolysis in an hypoxia/reoxygenation condition in order to counteract cell death in neurons (Ivanes et al., 2014). Another protective approach against ischemic insult consists in preconditioning the brain of animals that was partially protected when treated with neurotoxins, through activation of the Akt/p70S6K and PARP pathways, along with Bcl-xL (Formentini et al., 2014). However, it remains unclear whether these studies might be applied to patients and further investigations are needed to clarify the role of IF1 in age-related neurodegenerative disorders. Interestingly, the lack of IF1 induces a rare mitochondrial disease which is known as Luft’s disease and is characterized by high ATP hydrolysis, mitochondrial uncoupling, inefficient ATP synthesis and altered mitochondrial morphology (Dimauro et al., 1976; Yamada and Huzel, 1992). In this pathological condition the calcium amount which is accumulated in mitochondria is decreased suggesting the involvement of the permeability transition (Dimauro et al., 1976).

A crucial role of IF1 has been demonstrated in decreasing apoptosis and excessive microglial activity thus promoting neuronal and visual development of vertebrates (Martín-Jiménez et al., 2018). Increased apoptosis and neuroinflammation were shown in both retina and brain of a zebrafish model lacking one of the two IF1 paralogs, resulting in visual loss. The reduction in OPA1 level was described to be also associated to this process (Martín-Jiménez et al., 2018). Although these findings are in contrast with the lack of visual impairment in the first IF1 knockout mouse model (Nakamura et al., 2013), the lack of IF1 in neurons in a different mouse model caused impairment of cognitive functions (Esparza-Moltó et al., 2021). On the contrary, the overexpression of IF1 in mice increased learning and memory abilities (Esparza-Moltó et al., 2021). The authors explained these effects with the role of IF1 in mediating ATP synthase dimer stability and the mtROS-induced nuclear responses caused by IF1 effect on the ATP synthase activity (Esparza-Moltó et al., 2021). The known effects of IF1 on ATP synthase dimer stability and cristae organization might be protective for neurodegenerative diseases, since during brain aging and neurodegeneration many mitochondria undergo enlargement and structural disorganization (Moreira et al., 2010) often followed by altered mitochondrial function (Bosetti et al., 2002; Schmidt et al., 2008; Rossi et al., 2020, 2021). Another protective effect of IF1 might be inferred from pharmacological compounds that similarly inhibit ATP hydrolysis. Among different polyphenols binding directly to mitochondrial ATP synthase, resveratrol interacts with specific residues of α, *ß* and *γ* subunits at high concentrations by binding to the *γ* subunit and the βTP subunit (Gledhill et al., 2007), similarly to IF1. Resveratrol binding prevents both ATP synthesis and hydrolysis activities by blocking the rotation of *γ* subunit in mammalian ATP synthase (Gledhill et al., 2007). Resveratrol was shown to have neuroprotective effects in cerebral ischemia and other models of neurological disease, such as Parkinson’s, Alzheimer’s and Huntington’s (Sun et al., 2010; Kuršvietienė et al., 2016; Salehi et al., 2018), although the association of its protective effects and the interaction with ATP synthase requires further investigations.

Moreover, the proposed role of IF1 in promoting mitophagy also goes in the direction of a protective mechanism of the inhibitor protein, since the autophagic pathways are important for the maintenance of functional neurons, particularly in the context of mitochondrial and degenerative diseases (Moreira et al., 2010; Granatiero et al., 2016).

## Conclusion

The main role of the inhibitor protein IF1 is the protection from cell death in anoxia/near-anoxia conditions by limiting ATP dissipation. IF1 can also act as a pro-survival protein in cells exposed to severe respiratory chain damage. In addition, other mechanisms of action of IF1 have been proposed that may be relevant in the pathophysiology of cancer and neurodegenerative diseases including mitochondrial cristae organization, mitophagy and resistance to apoptosis. Although some of these IF1 functions need further investigation, they might represent a promising pharmacological target for cancer and neurodegeneration in the future.
